# Of Vital Action in Animals, and of the Influence of the Atmosphere

**Published:** 1842-07-23

**Authors:** 


### MEDICAL EXAMINER.

NEW SERIES.

No. 30.] PHILADELPHIA, JULY 23, 1842. [Vol. I.

### BIBLIOGRAPHICAL NOTICE.

*De l'Acte Vital dans les Animaux, et de l'Atmosphere.*

Par J. Liebig.

*Of Vital Action in Animals, and of the Influence of the Atmosphere.*

By J. Liebig.

[Journal de Pharmacie, vol. 1, Nos. 1 and 2, for March and April, 1842, p. 192. From Annalen der Chemie und Pharmacie, vol. xli, page 189.]

That the science of life will be largely the gainer by the recent progress of Organic Chemistry there can be, we think, but little doubt. Many intricate processes of the animal economy which, hitherto, as a cloak to our ignorance, we have been accustomed to call vital, will ultimately be found to depend on the operation of laws unequivocally chemical and physical. Others again are under the control of agencies entirely distinct from those which influence inert matter, and of whose intimate nature we will ever probably remain in ignorance, and whose laws will be the general expression of their phenomena, or of the conditions under which these take place. It must be borne in mind that organic chemistry, as a science, is yet in its infancy, and that it must mature by parts, if a vigorous manhood is expected. Many of its results will, doubtless, hereafter be found defective, for its laws are yet but imperfectly known; but this we conceive to be no argument against its steady prosecution. Its ultimate haven is usefulness. We fear more injury to it from its injudicious admirers than from its avowed enemies. The claims of the former are too eager, too presumptuous—disappointment may induce apathy, and finally disgust. Its own strength and merits are a sufficient protection against the attacks of the latter. Among its ardent and successful cultivators Professor Liebig certainly stands prëeminent. In his treatise on Agricultural Chemistry, he announces a similar work on Animal Physiology. Many of the prominent ideas of this were, it seems, embodied in a lecture delivered at Giessen, in the winter of 1840,–'41, and have since been widely promulgated by his auditory on that occasion. It is, therefore, to assert his claims to priority, that Professor Leibig has made the present publication. We propose to present our readers with a brief outline of his novel views.

The ingestion of aliment and the absorption of oxygen are the essential conditions of life. According to Lavoisier, an adult man receives in the course of one year 746 pounds of oxygen, and that without any appreciable change in his weight. This oxygen is eliminated in combination with carbon and hydrogen, forming carbonic acid and watery vapour. Admitting, with Lavoisier and Seguin, that an adult man absorbs daily sixty-five and a half ounces of oxygen, (46037 cubic inches=15661 grains by weight,) and that we estimate the mass of the blood at twenty-four pounds, with eighty per cent. of water, it results, from the known composition of the blood, that for the complete transformation of its carbon and of its hydrogen into carbonic acid and water, 66040 grains of oxygen would be required in four days and five hours. It is necessary, therefore, to introduce sufficient oxygen and carbon into the body to make up for the loss, and this is done by means of the food. An adult who takes ordinary exercise, consumes 27.8½ ounces of carbon per day, and this is disengaged, by means of the lungs and skin, in the form of carbonic acid gas. Now, for its transformation into carbonic acid, these 27.8½ ounces of carbon require seventy-four and a half ounces of oxygen. As no portion of the absorbed oxygen leaves the body in any other form than in combination with carbon and hydrogen, and, moreover, as the carbon and hydrogen eliminated are again replaced by carbon and hydrogen, which is introduced with the food, it is clear that *the nourishment necessary to the animal organism for its preservation, is in direct ratio with that of the oxygen absorbed.* Two animals who absorb in a given time, by the lungs and skin, unequal portions of oxygen, consume, in like proportion, unequal weights of the same food. The consumption of oxygen in a given time may be represented by the number of respirations; it follows, then, that in one and the same animal, the quantity of necessary aliment varies according to the force and number of the respirations. An infant, whose respiration is active, requires nourishment more frequently, and in larger proportional quantity, than an adult, and is, therefore, less able to support hunger. A bird, in whom the respiratory function is active, dies on the third day of hunger; a serpent, whose respiration is most sluggish, lives more than three months without food. The number of respirations in a state of repose and of action varies; the amount of necessary nourishment in the two states should be in proportion. Excess of nourishment, and a deficient supply of oxygen, great movement and feeble digestive organs, are incompatible.

The quantity of oxygen absorbed by the lungs does not depend alone on the number of respirations, but also on the temperature of the respired air. The capacity of the thoracic cavity is fixed, and at each inspiration a certain quantity of air enters, which may be considered as at a fixed volume; but its weight, and that of the oxygen which it contains, varies. Air is rarified by heat; condensed by cold. An equal volume of hot air and of cold air contains unequal portions of oxygen. If an adult absorb, at 25° C., 46037 cubic inches of oxygen, its weight would be sixty-five and a half ounces. In the inspiration of the same amount of oxygen, at 0° C., there are seventy cubic inches absorbed in the same time. In summer and in winter, at the equator and at the pole, we inspire a given volume of air; and if, in a given number of inspirations, we absorb, in summer, sixty-five and a half ounces of oxygen, the quantity absorbed at 0° rises to seventy. It is fifty-seven in Sicily at 35°, and seventy-two at — 10° The oxygen is eliminated after having undergone the same change in summer as in winter. At a low temperature we expire more carbon than we do at a high temperature, and consequently our food should contain proportionally more carbon in Sweden than in Sicily. In our temperate latitudes we should thus consume one-eighth more in winter than in summer. Such, indeed, is the order of nature; for the fruits which serve for nourishment to the inhabitant of the tropics, do not contain, when fresh, more than twelve per cent. of carbon, whilst the fat and whale oil, consumed by the inhabitant of the polar regions, contain from sixty-six to eighty per cent.

The reciprocity of action between the principles of the elements, and the oxygen disseminated throughout the body by the circulation of the blood, is the source of the animal heat. All living beings, whose existence depends on the absorption of oxygen, have a source of heat independent of the surrounding medium; and it is only those parts of the body accessible to arterial blood, and through it to the oxygen absorbed during the act of respiration, that maintain their own heat. The hair, wool, and feathers, have not an independent temperature. This elevated temperature of the animal body is everywhere, and in every circumstance, the result of the combination of a combustible substance with oxygen. The carbon of the food developes, in transforming itself into carbonic acid in the animal economy, the same amount of heat as if the combustion took place in the air, or in oxygen. It is clear that the quantity of oxygen, introduced at given periods by the act of respiration, ought to augment or diminish the amount of caloric liberated. Animals who have a lively and rapid respiration, and who consequently consume much oxygen, have a higher temperature than those who absorb less; an infant (39°) more than an adult, (37°.5;) a bird more (40—41°) than a quadruped, (37—38°;) and this last more than a fish or amphibious animal, whose temperature is only 1½° to 2° above the surrounding medium. Observation shows, that in all climates the temperature of man and of all warm blooded animals never varies, in spite of the immense difference in their modes of existence. The animal body is a warmed body, and observes the same relation to the surrounding medium as warm bodies in general, and receives heat if the exterior temperature is higher, and gives it if it be lower. The temperature of Palermo is the same or nearly the same as the average temperature of the body; but at the polar regions it is lower by 40° to 50°, and yet the temperature of the inhabitant of the two places is identical. This fact proves that the loss of heat is repaired immediately in the animal body, and this more rapidly in winter than in summer, at the pole than at the equator. Now in different climates, the quantity of oxygen that enters the body in respiration varies according to the temperature of the atmosphere; the quantity of inspired oxygen augments with the loss of air by cooling; that of the carbon and hydrogen, necessary for combination with this oxygen; ought to augment in a similar proportion.

It is clear that the reparation of heat is due to the reciprocal action of the principles of the elements, which become combined with the inspired oxygen. Whatever form the food takes in the body; whatever changes it undergoes, the last is the transformation of its carbon into carbonic acid, and of its hydrogen into watery vapour. The azote and the carbon not consumed by the oxygen, are eliminated by the urine and the solid excrements. The food is the combustible. It is by the aid of a sufficient absorption of oxygen that we obtain the heat developed by its oxydation. In winter, by exercise in the cold air, where the quantity of oxygen inspired is augmented, the demand for food, rich in carbon and hydrogen, increases in the same proportion; and it is the satisfaction of this want that procures us the most effectual defence against severe cold. A starved man freezes; and it is known that the carnivorous animals of northern climates are much more voracious than those of more southern countries.

In the cold and temperate zones the air, which has a continued tendency to destroy our body, excites us to labour, and to proper efforts to enable us to resist that tendency; whilst in warm climates the exigencies to procure food are by no means so pressing. Our habits are the equivalents of our food; the hotter it is the less strong is the desire to eat, precisely because the loss of heat, the cooling, and the necessity for reparation diminish. If we went naked as the Indian, or, if we were at the chase and fishing, exposed to the same degree of cold as the Samoide, we could devour half a calf, and a dozen of candles to boot, drink in proportion the same quantity of brandy or of train oil, precisely because their proportions of carbon and oxygen serve to establish an equilibrium with the external temperature. The necessary quantity is then regulated by the number of respirations, and by the temperature of the air that we respire, and by the quantity of heat that we give to exterior bodies. All facts concur to prove the truth of this law of nature. The Neapolitan cannot, without permanent or temporary injury, consume in his aliment, more carbon and hydrogen than he expires; and no inhabitant of the north can expire a greater quantity of these elements than have been introduced in his aliment, if he is neither sick nor hungry. The Englishman sees with regret his appetite, which is so often a source of enjoyment to him, lost in Jamaica, and he, in fact, succeeds, by Cayenne pepper and other strong stimulants, in taking the same amount of food as in his own country. But the carbon introduced into his body (by the food) is not consumed ; the temperature of the air is too elevated ; and an enervating heat does not permit an augmentation of the respirations, (by movement and exercise,) and thus place the consumption in ratio with the quantity introduced into the economy. In opposition to this conduct, when in certain patients the digestive organs are weakened, or lose the power of reducing the aliments to a condition to combine with oxygen, they consequently produce less resistance than the climate and the temperature require, and England sends them to a southern country, where the quantity of oxygen inspired decreases in a great proportion, and an amelioration in the state of health ensues. The digestive organs, in a diseased state, have enough power to place the least quantity of aliment in relation with the oxygen employed ; in a climate less cold the respiratory organs should be subservient themselves to this resistance. In summer we have the diseases of carbon—of the liver ; the diseases of oxygen—of the lungs, in winter. The cooling of the body, from whatever cause, requires a greater quantity of food. Simply being in the air, in a carriage, or on the deck of a vessel, augments, by radiation and increase of evaporation, the loss of heat, without even any increase of movement. The same happens to those accustomed to drink large quantities of cold water; it increases the appetite ; and a delicate constitution ought to restore, by exercise to the body, the oxygen necessary to the repairing of the heat lost.

Singing, talking, damp air, exercise an appreciable and determinate action upon the quantity of aliment to be consumed. We have seen that it is principally the carbon and hydrogen which serve, in combination with oxygen, to the production of animal heat; but hydrogen does not play a less important part than carbon. We know the origin of the carbon and of the hydrogen, for we see them diminish in the body with the duration of hunger. The first effect of hunger is a disappearance of the adipose matter. Each day sixty-five and a half ounces of oxygen enters the body of a starving man, and carries off a portion of the fat in leaving it. Animals who sleep during winter, as well as the periodical accumulation of grease in others, which disappears at other periods of their life, are all well known facts, which prove that there is no selection on the part of the oxygen. It combines with all that is presented to it, and for want of hydrogen it combines with the carbon, because, at the temperature of the body, the affinity of the hydrogen for oxygen is greatly superior to that of carbon. We know, in fact, that herbivorous animals expire a volume of carbonic acid equal to that of the oxygen inspired, whilst the carnivora, the only class of animals whose nourishment contains fat, absorb a greater quantity of oxygen than corresponds to the volume of carbonic acid expired; positive experiments have shown that, in certain cases there is expired, in the form of carbonic acid, only one-half of the volume of oxygen. But among those who die of hunger, the fat alone does not disappear; all the solid and soluble substances are absorbed by degrees. In the bodies of individuals dead of hunger, the muscles are soft and flaccid, and have lost their contractility; all parts of the body which were capable of passing to an active state, have served to protect the rest of the organs against the destruction of the atmosphere. In the last place, the brain takes part in this general oxydation; from this results wandering, delirium, death; all resistance ceases: chemical action and putrefaction commence, and all the parts of the body combine with oxygen. The time in which a person starves to death is regulated by the degree of fat, the amount of exercise and work, the temperature of the air, and the presence or absence of water.

In all chronic diseases death is due to the same cause—the action of the atmosphere. If the substances destined in the organism to the maintenance of the respiratory act fail; if the organs of the patients refuse to perform their duties; if they lose the faculty of bringing, for their own defence, the ingested food to that state where its elements can combine with the oxygen of the air, their own substance, the fat, the brain, the muscles and nerves are employed for this purpose.

The real cause of death in these cases is in the respiratory act—the action of the atmosphere. Want of nourishment, want of the faculty to assimilate to the organism, is want of resistance; it is the negative cause of the cessation of the vital activity. The flame is extinguished because the oil is consumed; the oxygen of the air has consumed it.

In certain states of disease, certain non-assimilable substances are produced; the simple abstinence from food eliminates them from the body; they disappear without leaving any trace,—their elements combining with the oxygen of the air.

From the moment that the function of the skin or of the lung experiences a perturbation, there appear in the urine substances rich in carbon, and it becomes of a dark brown colour. The respiration is the pendulum, the cause of movement; the respirations are the oscillations of the pendulum, which regulate it.

The practical bearing of many of the foregoing views will, no doubt, be fully appreciated by most of our readers. In his Theory of Animal Heat, Professor Leibig seems to incline to the Chemical School. We think that, from the most recent inquiries on this subject, we should look to the organic processes as its source, and that its generation and maintenance is due in a great measure to the molecular changes which occur in the systemic capillaries, and that though influenced by the nervous system, it is not dependent upon it. The late experiments of Bischoff and Magnus satisfactorily prove that the union between the oxygen and carbon occurs in the course of the circulation, and that it is there that the blood acquires its higher temperature. In plants, it has been shown that the amount of caloric evolved is in direct proportion to the activity of the vital processes, and attains its maximum at the periods of germination and flowering. According to Dr. Carpenter, the sexual apparatus of the Arum Italicum has been found to consume 132 times its own bulk of oxygen in twenty-four hours.

The experiments of Dulong and Despretz indicate, unequivocally, another source of animal heat besides the respiration; for they found that the caloric derived from the union of the carbon and hydrogen amounted, in herbivora to seven-tenths, and in carnivora to one-half only of the whole heat developed.

That the evolution of caloric in animals is dependent on certain chemical alterations occurring in the living system, and that the amount liberated observes a constant relation to the activity of these functions, derives interesting confirmation from the observations of Mr. Newport, on the development of heat in the Insect Tribe. In their larva state they have no independent heat, their temperature fluctuating with that of the surrounding medium, and their respiratory apparatus is very imperfectly developed. In the perfect insect, where the organs of respiration are largely developed, the temperature of the body frequently rises 10° to 15° F., above the air. The volant insects, who have the largest respiratory apparatus, have the highest temperature.

That the cutaneous surface in the higher animals is still an aërating apparatus, and contributes in a great degree to the maintenance of the animal heat, has been shown by the investigations of Breschet and Becquerel. They removed from rabbits their hair, and coated them over with a composition composed of glue, suet and resin, impermeable to air. The temperature of one rabbit, which was 100° F., before the operation, fell, when the material was dry, to 89½° F., and in the course of an hour to 76° F. In another animal, when the plaster was completely dry, the temperature fell to 67° F., and it died in the course of an hour. When the function of the lungs is materially impaired by organic disease, how far the cutaneous surface becomes a vicarious organ, and in what degree the phenomena of hectic are involved in this compensatory action, would, it strikes us, be an interesting and legitimate object of inquiry. M. C.

---

### THE MEDICAL EXAMINER.

PHILADELPHIA, JULY 23, 1842.

Some months since we published an advertisement extracted from a newspaper, purporting that the author had discovered a new method of treating strictures of the urethra, and warranting a cure even in the worst cases. We then thought it our duty to express our disapprobation of such advertisements, in which we are pleased to find that we are sustained by our cotemporary, the American Journal, as well as by the general feeling of the profession. We at the same time stated, that we regretted that the advertisement came from a surgeon of eminence, whose example was, therefore, of no little importance.

Within a few days we have received a letter from Dr. J. P. Mettauer, of Prince Edward Court House, Virginia, the author of the advertisement; it is written in a manly and gentleman-like tone, and we are very happy to state the reasons which determined the author to this unusual course. He has not made his method a secret, but has freely communicated it to his professional friends, and is at this time preparing a memoir and course of lectures upon the subject. But his investigations and his instruments were not at the time complete, and he was unwilling to lose the merit of the discovery, which would have been placed at the mercy of persons who might abuse the information.

We are also glad to state our belief that Dr. Mettauer is perfectly free from the suspicion of concealing a method of treatment for the sake of gain; but although he was actuated by the reasons which he has assigned, we still think that all advertisements warranting cures, even by means of old and well known methods, are wrong, and that Dr. Mettauer has taken a view of the subject which is erroneous, and at variance with the general feelings and practice of the profession. An easy mode of testing the matter is to extend the application of the principle. Many persons imagine, on much less valid grounds than Dr. Mettauer, that they possess peculiar skill in the treatment of different diseases, and if they are sustained by the example of men eminent in the profession, they would eagerly seize the opportunity of making public their claims by newspaper advertisements. It is easy to imagine what the result would be, and what influence such a course would have upon the profession, as well as upon the public. For these reasons, the different medical associations in Europe and America have either expressly or tacitly discountenanced all advertisements of a nature similar to the one in question.

We have not the pleasure of a personal acquaintance with Dr. Mettauer, but we have many common friends, and his reputation as a skilful surgeon and able writer is well known to us; it is therefore with regret that we differ with him on a question of ethics. We shall not, however, be the less pleased to communicate the results of his researches to our readers as soon as they are published, and hope that they will give us much new and solid information as to the treatment of a most troublesome class of disorders—the diseases of the urinary organs.

